# Epigenetic Alterations of Maternal Tobacco Smoking during Pregnancy: A Narrative Review

**DOI:** 10.3390/ijerph18105083

**Published:** 2021-05-11

**Authors:** Aurélie Nakamura, Olivier François, Johanna Lepeule

**Affiliations:** 1 Université Grenoble Alpes, Inserm, CNRS, IAB, 38000 Grenoble, France; johanna.lepeule@univ-grenoble-alpes.fr; 2 Université Grenoble Alpes, Laboratoire TIMC, CNRS UMR 5525, 38000 Grenoble, France; olivier.francois@univ-grenoble-alpes.fr

**Keywords:** DOHAD, epigenetics, DNA methylation, tobacco smoking, pregnancy, mediation, cell heterogeneity, review

## Abstract

In utero exposure to maternal tobacco smoking is the leading cause of birth complications in addition to being associated with later impairment in child’s development. Epigenetic alterations, such as DNA methylation (DNAm), miRNAs expression, and histone modifications, belong to possible underlying mechanisms linking maternal tobacco smoking during pregnancy and adverse birth outcomes and later child’s development. The aims of this review were to provide an update on (1) the main results of epidemiological studies on the impact of in utero exposure to maternal tobacco smoking on epigenetic mechanisms, and (2) the technical issues and methods used in such studies. In contrast with miRNA and histone modifications, DNAm has been the most extensively studied epigenetic mechanism with regard to in utero exposure to maternal tobacco smoking. Most studies relied on cord blood and children’s blood, but placenta is increasingly recognized as a powerful tool, especially for markers of pregnancy exposures. Some recent studies suggest reversibility in DNAm in certain genomic regions as well as memory of smoking exposure in DNAm in other regions, upon smoking cessation before or during pregnancy. Furthermore, reversibility could be more pronounced in miRNA expression compared to DNAm. Increasing evidence based on longitudinal data shows that maternal smoking-associated DNAm changes persist during childhood. In this review, we also discuss some issues related to cell heterogeneity as well as downstream statistical analyses used to relate maternal tobacco smoking during pregnancy and epigenetics. The epigenetic effects of maternal smoking during pregnancy have been among the most widely investigated in the epigenetic epidemiology field. However, there are still huge gaps to fill in, including on the impact on miRNA expression and histone modifications to get a better view of the whole epigenetic machinery. The consistency of maternal tobacco smoking effects across epigenetic marks and across tissues will also provide crucial information for future studies. Advancement in bioinformatic and biostatistics approaches is key to develop a comprehensive analysis of these biological systems.

## 1. Introduction

### 1.1. The Burden of Maternal Tobacco Smoking during Pregnancy

In Western countries, the prevalence of maternal tobacco smoking during pregnancy is estimated to be 8%, including around three-quarters of daily smokers, and with a high heterogeneity between countries [1]. Hence, despite an overall decrease in trends in tobacco smoking [2], the prevalence of tobacco use remains elevated in women in the general population (e.g., 30% in France, 25% in Germany, 16% in the United Kingdom, or 15% in the United States of America) [3] and in pregnant women (e.g., 16% in France, 9% in Germany, 12% in the United Kingdom, or 7% in the United States of America) [4,5,6]. According to a nation-wide study conducted in a representative population of women giving birth in France in 2016 [7], among women who smoked before pregnancy, 46% managed to quit during pregnancy and 45% reduced their consumption of tobacco. For the last 9% of women, their consumption of tobacco remained as it was prior to pregnancy. In comparison to other pregnant women, women smoking tobacco during pregnancy were more likely to be addicted to nicotine, to have a partner also being smoking tobacco, and to have a lower socioeconomic position [4,7], which is associated with further negative issues related to pregnancy outcomes and to offspring [8,9,10]. Furthermore, rates of maternal tobacco smoking during pregnancy are likely underestimated, especially in the most deprived areas, due to social and medical pressure on smoking pregnant women [11]. Therefore, maternal tobacco smoking during pregnancy remains an important issue in public health and the leading preventable cause of birth and offspring complications [12].

### 1.2. Effects of Maternal Tobacco Smoking during Pregnancy on Pregnancy Outcomes

The burden of tobacco smoking is particularly heavy on the pregnant women population, as smoking is linked to numerous adverse health outcomes for both the mother and the developing child [13]. Metabolism of nicotine was shown to be higher in pregnant women than outside of pregnancy, with higher risks of having symptoms of withdrawal [14]. Women smoking tobacco during pregnancy are more likely to experience pneumonia, influenza, bronchitis, and myocardial infection than non-smoking pregnant women. They are also more likely to experience pregnancy complications such as ectopic pregnancy, placenta prævia, intrauterine growth restriction, and delivery issues including preterm birth [15,16,17,18,19]. However, maternal tobacco smoking during pregnancy has been consistently associated with lower risks of pre-eclampsia, probably through vascular protective effects of carbon monoxide [18].

### 1.3. Effects of Maternal Tobacco Smoking during Pregnancy on Early and Later Offspring Phenotypes

Regarding the developing child, effects of maternal smoking are mainly related to the growth of the fetus, the development of the respiratory and neurological systems of the child, and later risk of substance abuse [20,21]. Many studies have shown that in utero exposure to maternal tobacco smoking was associated with smaller birth weight and head circumference, and reduced length for gestational age, with a dose–response relationship [8,10,22,23]. However, one study based on 677,922 singletons identified from the Swedish Medical Birth Register, including 62,941 siblings born from 28,768 mothers, showed the effect of maternal tobacco smoking during pregnancy on birthweight reduction was less marked in the sibling analysis than in the conventional analysis, meaning that part of this association could be explained by genetic characteristics [24]. Later in life, maternal tobacco smoking during pregnancy is associated with an increased risk of their child being overweight or obese [25,26,27]. Children of mothers who smoked tobacco during their pregnancy tend to have reduced lung function and a slower development of immune system, which are associated with an increased risk of respiratory infections [28,29]. Impairment in child’s neurodevelopment is associated with maternal tobacco consumption during pregnancy with higher risk of attention-deficit hyperactivity disorder (ADHD) [30,31], behavioral and conduct problems [32,33], as well as difficulties with learning, memory, and academic achievement [34,35]. Longitudinal studies also found that children exposed to maternal prenatal tobacco smoking were more likely to have long-term effects on cardiovascular factors [36] and to experience later substance abuse [37]. However, studying the effects of maternal tobacco smoking during pregnancy on later offspring health and development is challenging, as analyses need to be adjusted on a large number of potential confounders (e.g., familial, socioeconomic, psychosocial factors, preexisting health disorders in the mother and father) [37,38]. For example, in a twin study, maternal tobacco smoking during pregnancy was significantly associated with ADHD in children, but genetic factors explained a greater part of the variance in offspring ADHD than exposure to maternal tobacco smoking during pregnancy [39,40].

A few studies also found evidence that grandmaternal tobacco smoking during pregnancy could have multigenerational effects, by estimating increased risk of asthma in grandchildren, independently of their mother’s smoking status [41,42,43].

### 1.4. Toward Understanding of Underlying Mechanisms: The DOHaD Hypothesis

The large research corpus on short- to long-term health effects of maternal tobacco smoking during pregnancy echoes the work conducted within the developmental origins of health and disease (DOHaD) field of research, which explores the links between the early life environment and the risk of chronic diseases from childhood to adulthood. The DOHaD theory was formulated initially by David Barker in the 1990s [44,45,46]. The first 1000 days of life [47] from conception, and especially gestation, is a critical period of development where organs are simultaneously developing and vulnerable to environmental insult [48,49]. When exposed to stressful conditions (such as famine or exposure to pollutants), the fetus might have to adapt and regulate himself in order to survive [50]. Epidemiological and animal studies suggest the existence of critical windows of vulnerability, including in utero and early postnatal periods. Thus, exposure to tobacco smoke during these critical windows of development could be more harmful than during other periods with regard to early and later child health and development [51,52]. On the other hand, these critical windows can also be seen as opportunity windows that could be targeted by public health policies [53]. In fact, pregnancy being a period of changes could provide opportunities to intervene on modifiable factors such as tobacco smoking [23]. Such early and timely interventions to reduce tobacco smoking will likely have a large impact on offspring’s later health [54].

The phenomena of long-term health effect of early life exposure refer to developmental programming. A few underlying mechanisms of developmental programming have been proposed. They include excessive exposure of the fetus to glucocorticoids, dysregulation in the development of the hypothalamic–pituitary–adrenal (HPA) axis [55], irreversible changes in organ structure, and alterations in epigenetic mechanisms such as DNA methylation (DNAm), micro-RNAs (miRNAs), and histone modifications. Epigenetic mechanisms, through developmental programming could mediate the observed associations between environmental exposures, such as maternal smoking during pregnancy, and later offspring health outcomes. Epigenetic marks are involved in the structure of chromatin and in gene expression regulation [56]. They can also be partly transmitted to descendants [51,57].

In addition to their potential mediating effects of environmental exposures on health outcomes, epigenetic marks can also be seen as an adaptive mechanism. In this latter case, there are adaptive changes in response to the environment, but without adverse health outcome, while in the first case, there are maladaptive changes that lead to an increased risk of disease [58]. Epigenetic marks can be used as exposure biomarkers, as recently shown for in utero cigarette smoke exposure using DNA methylation measured in adolescents’ blood [59], and as health biomarkers to predict disease risk or progression. Reliable markers of in utero tobacco exposure would be an important epidemiological advance as it would help identifying the long-term health impact of in utero tobacco smoke exposure on diseases developing slowly over an extended period of time (cancer, respiratory and heart diseases, etc.). Ultimately, health biomarkers might be used to detect physiological disturbances before the patient presents the disease, in order to provide timely medical support. Additional research in this area might also lead to the development of therapeutics that target epigenetic processes. In epigenetic epidemiology applied to the DOHaD, maternal smoking is one of the exposures that has been the most widely investigated. A growing number of studies have characterized the associations between maternal tobacco smoking during pregnancy and epigenetic mechanisms [19,21,36,51,59,60,61,62,63,64,65,66,67,68,69,70,71,72,73,74,75,76,77,78,79,80,81,82,83,84,85,86,87,88,89,90,91,92,93,94,95,96,97,98,99,100,101,102,103], using different methodological approaches and different tissues (including placental, cord blood, blood, etc.). Most studies rely on DNA methylation, which is the best understood and the most cost-effective epigenetic mark to consider in epidemiological studies, and it is conducted on placenta, cord blood, or peripheral children’s blood. Extensive reviews have been published in the last couple of years, including specific reviews on maternal tobacco smoking during pregnancy and epigenetics [58,104,105] and more general reviews on smoking and epigenetics [106,107,108].

The purpose of this review was to provide an update of the main results from epidemiological studies on the impact of in utero exposure to maternal tobacco smoking on epigenetic mechanisms. Then, we focused on a few methodological issues arising in such studies, including the tissues investigated and the methods used to analyze epigenetic data, mainly DNA methylation.

Inclusion criteria were original research studies and reviews focusing on maternal tobacco smoking during pregnancy and epigenetics. Exclusion criteria included animal studies, electronic cigarette smoking, and cannabis consumption. Databases such as PubMed and Google Scholar were non-systematically searched, as well as references from the retrieved articles. No time frame or language criteria were applied.

## 2. Description of Epigenetic Regulation Mechanisms

In this section, we survey DNA methylation, miRNA, and histone modification mechanisms and summarize the principal results of epidemiological studies regarding the association of maternal tobacco smoking during pregnancy with each epigenetic mechanism.

### 2.1. DNA Methylation

DNA methylation is defined as a chemical process by which a methyl group is added to a specific cytosine base of DNA and then converted to a 5-methylcytosine by DNA methyltransferase enzymes. Most of DNA methylation occurs on cytosines that precede a guanine nucleotide (also called “CpG sites”) [109]. DNA methylation does not change DNA sequences of nucleotides; it can be replicated through cell divisions and can be either reversible or persist via biological memory [97,110]. The implication of DNA methylation in gene expression regulation was demonstrated as early as 1980 [111,112]. The methylation of a CpG island, defined as a region with a high frequency of CpG sites, combined with irregular binding of a variety of proteins, may result in blocking the transcription of coding RNA into proteins. Especially, when a CpG-rich promoter region is highly methylated, the gene will be more likely to be silent and not expressed. On the contrary, when the gene’s promoter region is hypomethylated, the gene will be more likely to be expressed. DNA methylation in other regions of a gene can have a different effect on gene expression [113,114].

DNA methylation can be altered by environmental factors (reviewed by Alvarado-Cruz and colleagues and [115] and Vlahos and colleagues [116]) ranging from diet to maternal stress and to pollutants such as arsenic, persistent organic pollutant, endocrine disruptors, air pollutants [117,118], and tobacco smoke, especially during the earlier stages of the child’s development as the epigenome undergoes considerable reprogramming during gametogenesis and the preimplantation embryonic stage [119]. Developmental programming continues during organogenesis through a remarkable plasticity of the epigenome during the developmental period. Therefore, environmental insults during this period could alter DNA methylation patterns.

Exposure to maternal tobacco smoking during pregnancy has been shown to be associated with DNA methylation changes in different tissues, both using gene-candidate and genome-wide approaches. More detailed results were summarized in previous reviews (e.g., [58,82,91,107,120]). Briefly, in neonates, DNA methylation was mostly measured in cord blood [61,74,76,78,85,88,121,122] and placenta [64,87,97,123,124,125]. Using an epigenome-wide association study (EWAS) approach, Joubert et al. [74] found that maternal tobacco smoking during pregnancy was associated with differential cord blood DNA methylation in the AHRR, CYP1A1, and GFI1 genes. Meta-analyzing cord blood samples from 13 birth cohorts from the Pregnancy and Childhood Epigenetics (PACE) consortium, Joubert and colleagues found that over 6000 CpGs were differentially methylated in relation to maternal smoking at genome-wide statistical significance, including close to 3000 CpGs located in 2000 genes that had not been related to maternal tobacco smoking during pregnancy before [76]. Reese and colleagues [93] were able to identify a biomarker of maternal smoking during pregnancy in newborns, using cord blood DNA methylation data. In studies conducted by Suter and colleagues [124,125], children exposed to maternal tobacco smoke during pregnancy showed significant placental DNA hypomethylation and increased placental CYP1A1 expression in comparison to non-smoking mothers. Using an EWAS approach, Morales and colleagues identified 50 CpGs differentially methylated in placentas exposed to maternal tobacco smoking during pregnancy [87]. The authors found a dose–response relationship in cg27402634, located between genes LINC00086 and LEKR1, which was previously established as being associated with birthweight in genome-wide studies, and that 36% of the effects of maternal tobacco smoking during pregnancy on offspring’s birthweight could be mediated by cg27402634’s methylation levels. Another study found that placental DNA methylation at seven CpGs, located near genes MDS2, CYP1A2, VPRBP, WBP1L, CD28, CDK6, and PBX1 (PBX1 being involved in skeletal patterning and programming), was mediating the relationship between maternal tobacco smoking during pregnancy and offspring’s birthweight [64]. In a recent EWAS investigating placental DNA methylation in 568 pregnant women, either actively smoking during their pregnancy, formerly exposed to tobacco smoking, or not exposed to tobacco smoking during pregnancy [97], the authors identified 152 differentially methylated regions (DMRs) with “reversible” alterations of DNA methylation, which were only present in the placenta of current smokers, whereas 26 DMRs were also found altered in former smokers who had quit smoking prior to pregnancy and whose placenta had not been exposed directly to cigarette smoking. The authors also showed that the 203 tobacco-induced DMRs identified were significantly enriched in epigenetic marks corresponding to enhancer regions and in regions controlling the monoallelic expression of imprinted genes. These data suggest that tobacco smoking during pregnancy could impact the transcription of genes normally regulated by mechanisms involving DNA methylation as well as how it could affect the development and growth of the fetus.

Prenatal exposure to maternal tobacco smoke was also associated with significant changes in DNA methylation either in buccal cells [62,99], blood cells [36,63,68,69,80,95,101,126], or in specific tissues such as fetal lung [65]. A study conducted on tissues from the cortical plate of fetal brains identified DMRs between exposed and unexposed fetuses to maternal smoking. The results showed a global hypomethylation in 20% of the exposed fetuses, with the most hypomethylated regions located in the SDHAP3 and GNA15 promoters. These smoking-induced changes in DNA methylation led to a decreased number of neurons in fetal brains and alterations in cell-type differentiation [127]. DNA methylation alterations due to maternal smoking were shown to be greater in male offspring than in females [88,122], suggesting a sex-specific effect that might further help understanding sex-dependent susceptibility to diseases.

Most studies have investigated the impact of maternal smoking during pregnancy on DNA methylation using cross-sectional designs [80,101]. A few recent studies have used a longitudinal approach, either using several time points in exposure assessment or in DNA methylation, trying to identify the persistence of tobacco-induced DNA methylation changes [36,95]. In the Avon Longitudinal Study of Parents and Children (ALSPAC), where DNA was extracted from cord blood samples collected at birth and from blood samples collected in children at ages 7 and 17, longitudinal analyses highlighted the persistence of altered DNA methylation patterns across ages associated with in utero exposure to maternal tobacco smoke [94]. Similar results were shown in the Peri-postnatal Epigenetics Twin Study (PETS) [90] and in the Lifestyle and environmental factors and their Influence on Newborns Allergy risk (LINA) cohort using whole genome sequencing [128].

Whereas a dose–response relationship was underlined by several studies (the more pregnant women smoked, the greater were changes in DNA methylation) [86,103], a recent study suggests that any exposure to tobacco smoke during pregnancy could be harmful toward infant lung function [99]. Monasso et al. [86] and Rousseaux et al. [97] explored different timing of exposure around pregnancy. In cord blood, Monasso et al. identified 1391 CpGs differentially methylated in sustained (but not in women quitting smoking prior or in early pregnancy) vs. never smokers, among 5915 CpGs established with robust evidence for association with sustained maternal smoking in a prior meta-analysis [76]. They concluded that quitting smoking during pregnancy might be associated with DNA methylation at other CpGs [86]. In accordance with these conclusions, the results of the EWAS performed by Rousseaux et al. on former, current, and non-smokers during pregnancy identified specific CpGs/DMRs in the placenta associated with these smoking statuses, suggesting the establishment of a memory of exposure to tobacco affecting the methylation pattern of placentas that had never been directly exposed to smoking [97].

To conclude, a growing number of studies have identified differentially methylated CpGs in different tissues or blood in offspring exposed vs. non-exposed to maternal smoking during or prior to pregnancy, either using gene candidate or EWAS approaches. Technical and methodological challenges of these analyses are discussed later in this review.

### 2.2. miRNAs

Micro-RNA (miRNA) are defined as a group of small endogenous non-coding RNAs with a length of approximately 22 nucleotides that play an important role in gene expression by regulating developmental and cellular processes (cell proliferation, differentiation, and apoptosis, or cell death) [129,130] and by binding targeted mRNA (translation repression) and altering its protein expression [131]. MiRNA, as DNA methylation, could act as a potential mark of environmental insults for fetuses during pregnancy [132,133].

In comparison to DNA methylation, only a few studies focused on the associations between maternal tobacco smoking during pregnancy and miRNA. In one of the first studies conducted on miRNA, Maccani et al. [83] analyzed the expression of four candidates (miR-16, miR-21, miR-146a, and miR-182) implicated in growth and developmental processes, measured in placentas with regard to exposure to maternal smoking during pregnancy and specific components of cigarette smoke. Despite a small sample size (25 placentas), three of them (miR-16, miR-21, and miR-146a) turned out to be significantly downregulated in placentas exposed to tobacco smoke in comparison to non-exposed placentas [83]. In cord blood, Herberth et al. established an association between maternal tobacco smoke during pregnancy and miR-223 expression, which could be involved in offspring’s risk of allergies later in life [70]. In a recent study conducted on 1200 children included in the Human Early Life Exposome (HELIX) project, a consortium compounded by six European birth cohorts, the authors investigated the associations of maternal smoking during pregnancy and childhood secondhand smoke exposures with changes in four layers, including blood DNA methylation and plasma proteins, sera and urinary metabolites, and gene and miRNA expression [103]. They found that maternal tobacco smoking during pregnancy was associated with DNA methylation changes at 18 loci in child blood but not with the expression of the nearby genes. The authors also found dose–response trends with higher dose or duration of exposure in smoking-related molecular marks. Interestingly, a weaker association between maternal tobacco smoking during pregnancy and gene expression in comparison to DNA methylation was found in former smokers, suggesting different reversal rates after smoking cessation and a methylation-based memory to previous exposures [103]. Finally, the results of a few studies suggest that dysregulation in miRNA expression due to maternal tobacco smoking during pregnancy could have multigenerational effects [38,70,107,134].

The lack of studies and lack of consistency in miRNA changes identified to be associated with maternal tobacco smoking during pregnancy in comparison to DNA methylation results could be explained by the higher instability of the RNA in comparison to DNA [103].

### 2.3. Histone Modifications

Histone modifications are defined as chemical modifications influencing gene expression. They can be either inducible and promoting gene expression or repressive, depending on their influence on chromatin packaging [109]. Few epidemiological studies have been able to assess the effect of exposure to maternal tobacco smoking during pregnancy on histone modification because of technical challenges.

On the LINA mother–child birth cohort, DNA was extracted from mother’s and children’s blood [128]. The authors explored the link between maternal tobacco smoke-associated DMRs and the status of the chromatin. They investigated two histone marks characterizing active chromatin (H3K4me1 and H3K27ac) and two histone marks characterizing a repressive state of the chromatin (H3K9me3 and H3K27me3), which covered about 4.0 and 3.8% of active states of the genome in children and mothers, respectively, and about 1.8 and 2.2% of repressive states, respectively. They identified more smoking-associated transitions to repressive states in mothers and more transitions to active states in children. Their results suggest that maternal tobacco smoking is associated with a hyperactive state of the chromatin in children. The authors conclude that the different responses of DNAm and histone modifications to tobacco smoking between children and mothers might be due to a higher exposure of the unborn child to some components of tobacco smoke compared to the smoking mother. In both mothers and children, about twice as many maternal tobacco smoking-related DMRs were located in enhancers than in promoters. These results echo those from Rousseaux et al. obtained in placental DNA methylation, showing an enrichment in H3K4me1 and H3K27ac marks among the tobacco-smoking associated differentially methylated regions, which pointed toward enhancer regions [97].

In a recent study conducted on rats, Xie and colleagues demonstrated that acetylation modifications at histone H3K9 of TGFβ, TGFβR1, SOX9, COL2A1, and ACAN gene promoters were significantly lower in the articular cartilage of pregnant female rats directly exposed to nicotine in comparison to non-exposed pregnant rats and that deacetylation at H3K9 of the TGFβR1 and COL2A1 gene promoters was transmitted to the next generation of rats [135].

These few results on histone modifications illustrate how a global approach including DNAm, histone modifications, and miRNA would provide a much more comprehensive picture of the effects of maternal tobacco smoking on epigenetic mechanisms and of our understanding of the potential health effects of such modifications.

## 3. Target Tissues and Proxies

Epigenetic marks, mostly DNA methylation, have been investigated in different tissues in association with maternal tobacco smoking in pregnancy. Relevant tissue might differ regarding whether the study aim is to identify a marker of exposure and/or health outcome. For example, when studying children neurodevelopment or respiratory health, brain or lung tissue/airway epithelium could be the most relevant biological samples to investigate. However, collecting such tissues in living humans is in most cases unethical and not feasible. A few studies have collected post mortem brain samples in elderly; however, such post mortem studies are subject to bias due to the cause of death [136]. Therefore, a key question relates to the relevance of peripheral tissues as surrogate markers of targeted tissues and more generally to the correlation of epigenetic profiles and alterations across tissues.

Regarding tobacco smoking exposure in general, buccal or nasal cells are among the most accessible and relevant samples to collect. Peripheral blood is also relatively accessible, although it is considered more invasive and subject to a lower acceptance than buccal or nasal brush. A study has shown associations of maternal smoking during pregnancy with DNA methylation in offspring buccal cells [62]. DNAm from peripheral blood from the children has also been largely associated with maternal smoking during pregnancy [63,68,84,90,94]. One study, comparing blood DNAm marks in newborns exposed to maternal smoking during pregnancy and adults exposed to personal smoking, found 3838 differentially methylated CpGs (corresponding to 743 genes) specific to newborns exposed to maternal smoking in pregnancy and 1709 differentially methylated CpGs common in newborns and adults respectively related to in utero exposure to tobacco smoking and their own smoking [137].

The large majority of studies investigating maternal smoking during pregnancy have relied on cord blood DNA methylation. Several studies found a lower cord blood methylation level and higher expression of AHRR gene in children exposed to maternal tobacco smoking during pregnancy [74,90,104]. One study tried to replicate the effect of the exposure to maternal tobacco smoke on cord blood mononuclear cells AHRR methylation using children buccal cells and placentas. Children exposed to maternal tobacco smoking during pregnancy showed a lower methylation at cg05575921 in buccal epithelium and placenta in comparison to cord blood cells (on average 35% vs. 80% methylation) on paired samples [90], providing evidence of tissue specificity for maternal smoking effects on AHRR methylation. Possible explanations include the expression of AHRR, which could be lower in the placenta than in cord blood, differences in cell composition between the different tissues, or other more complex interactions in the epigenetic machinery [104].

Some studies have investigated the correlation of DNA methylation across tissues. Substantial intra-individual differences in DNA methylation across placenta, cord blood, and saliva collected in early infancy have been shown [138]. Using a genome-wide approach, De Carli et al. found a poor correlation between placental and cord blood DNA methylation measurements [139], and more recently, Groleau et al. found tissue-specific DMRs associated with tissue specific biological functions [140]. These results suggest a high degree of specificity for these tissues.

In the DOHaD context and more generally in perinatal studies, the placenta represents a relevant tissue that may act as an exposure biomarker, which makes it a potential record of fetal exposures, and as a health biomarker due to its master regulator function of the fetal hormonal and endocrine milieu. The placenta plays a key role in fetal programming by supporting both the health of the mother and the development of the fetus. It conveys nutrients and oxygen to the fetus and regulates gas and waste exchanges as well as hormone interactions [141]. The placenta acts as a partial barrier and many chemicals, such as polycyclic aromatic hydrocarbons, can pass through and reach the fetus [142]. The placenta is a transient organ considered as an accurate “molecular archive” of the prenatal environment [129,143]. Placenta collected at birth does not allow measures of DNA methylation changes through pregnancy, but as cord blood, it presents the advantage of being easy to collect after birth [107], with a high acceptability rate. Placenta also requires few ethical concerns for its use in medical research [105]. The relationship between DNA methylation in placenta and other tissues will likely vary depending on the target gene, exact location, and timing of sampling. However, studies indicate that placenta-based DNA methylation is a relevant proxy for brain tissue [144] particularly with regard to the placenta regulated glucocorticoid and serotonin pathways, which are important factors for brain maturation and cognition [145]. The placenta has been described as the “third brain” [146] and regulates fetal exposure to cortisol through the expression of HSD11B2, whose methylation levels are strongly positively correlated with fetal cortex methylation [144]. As for respiratory health, a study investigating matched placental and lung tissues showed that common DNA methylation pathways are affected by in utero nicotine exposure [65]. Overall, these studies suggest that epigenetic profiling of placenta might be relevant proxy for brain or lung tissue when studying neurodevelopment or respiratory outcomes.

## 4. Data Analysis

The multiple technical and methodological challenges to address when investigating DNA methylation data make comparison between studies difficult. Below, we review some of the methods used to address these challenges.

### 4.1. The Role of Cell Subtypes Proportions

Beyond tissue-specificity, epigenetic marks are also highly specific to cell types in tissues. Therefore, part of DNA methylation levels measured are driven by the cell composition of the collected tissue sample. Until recently, it was generally assumed that DNA methylation variation due to cell heterogeneity should be removed in association studies, since cell heterogeneity may confound the relationship with the response variable [147]. For some tissues, such as cord or peripheral blood, cell composition is measured or estimated using references (reviewed by Titus et al. [148] and Bakulski et al. [149]). For other tissues, such as placenta, cell composition is more difficult to measure, and most studies have used the reference-free algorithm of Houseman et al. [147], with the limitation that such algorithm provides an estimate of placental heterogeneity rather than cell proportion estimates. The recent work of Yuan et al. provides a useful reference for estimation of cell composition of placental samples from placental DNA methylation data [150], and Scherer et al. reviewed the latest development of this expanding field of investigation [151]. Note that some authors have recently argued that correcting for cell heterogeneity should be carefully considered depending on the purpose of the study, and the debate on cell heterogeneity correction is ongoing [152,153].

### 4.2. Confounding Factors

Attentive selection of potential confounders is essential when associating exposure to maternal tobacco smoking during pregnancy with DNAm. Cell heterogeneity may not be the only source of confounding, and other confounders must be considered as well. Those factors sort into two groups: (1) observed factors, such as mother’s age or body mass index at birth date, sex of the child, socioeconomic status, etc. and (2) unobserved factors, such as residual batch effects and additional unmeasured confounding variables.

For example, socioeconomic status, which is highly correlated with maternal tobacco smoking during pregnancy and DNA methylation and should be taken into account in multivariate analyses, can be challenging for researchers as those characteristics of parents tend to be under-reported or under-collected in perinatal studies [154]. Other factors such as partner smoking or the use of other substances during pregnancy are correlated to maternal tobacco smoking during pregnancy and DNA methylation and need to be taken into account in order to distinguish the effects of maternal tobacco smoking during pregnancy on epigenetic changes from other factors. Technical effects due to the array design include batch, plate, and chip, which could be major predictors of methylation level. Some methods used for the quality control steps and normalization of the raw beta values from the Illumina chip allow correcting for such technical effects. However, such correction might not be sufficient to remove all these technical effects and often requires further adjustment for these covariates in the association analyses. Recent statistical methods such as surrogate variable analysis [155], latent factor mixed models [156], or omic-data-based complex trait analysis (OSCA) [157] implement corrections for unobserved confounders, and they can be used in conjunction with observed confounders to decrease false-positive rates in DNA methylation association studies.

### 4.3. Gene Candidate, Global, and EWAS Approaches

Three complementary approaches, pursuing different objectives, are used to identify DNA methylation changes associated with maternal tobacco smoking during pregnancy. First, the candidate approach allows verifying an a priori hypothesis of the effects of maternal tobacco smoking during pregnancy on one or several identified genes. For example, studies conducted by Suter and colleagues evaluated the effects of maternal smoking during pregnancy on placental CYP1A methylation and expression [124], while Appleton and colleagues focused on placental HSD11B2 methylation [123]. Another hypothesis-based approach consists of studying pathways involved between in utero tobacco exposure and epigenetic mechanisms through the use of tools such as Kyoto Encyclopedia of Genes and Genomes (KEGG) pathways [158]. Second, EWAS do not make any a priori hypothesis about genes or CpGs that could be affected by maternal tobacco smoking during pregnancy [63,87,125]. Such studies aim at generating new hypotheses. Most studies investigate genome-wide changes in DNA methylation using the Illumina Infinium Human Methylation beadchip arrays (27 k, 450 k, and now EPIC) [159,160], which still represent a relatively small proportion of the genome. The 450k beadchip array was enriched for promoter regions, while the new EPIC covers 90% of the CpGs from the 450 k and has been enriched in enhancer sites. To date, most studies exploring effects of maternal smoking have relied on the 450 k beadchip array. A few studies used different techniques such as whole genome sequencing [128], but this approach remains costly and needs a high degree of technical expertise, which makes it difficult to apply in large epidemiological studies. Finally, methylation can also be assessed at a global level using repetitive interspersed elements such as LINEs and SINEs, which are considered as markers of genome stability.

### 4.4. Underlying Challenges of Multiple Testing

With the advent of high-throughput screening technologies, the number of CpGs has grown exponentially in epigenetic association studies. Statistical analyses usually test the association between each CpG and maternal smoking exposure, which imply a high number of tests, especially in the EWAS cases, but also in candidate-gene approaches depending on the number of candidates tested. Studying associations between maternal tobacco smoking during pregnancy and epigenetic mechanisms using a set of a priori selected CpGs or an EWAS approach involves multiple comparisons, and thus, significance values need to be corrected in order to minimize false discoveries [161]. Different methods for estimating a false discovery rates (FDR) were suggested by Benjamini and Hochberg [162,163] and Storey and Tibshirani [164,165], among others. Those FDR control approaches make rather strong assumptions on the null hypothesis and on the independence of the tests, which may lead to overly conservative decision thresholds.

While the number of CpG markers has increased exponentially, the number of participants in association studies has remained relatively low. Therefore, statistical power may be an issue, which is worsened by the low amount of absolute variation in DNA methylation associated with maternal smoking, correlation among CpGs, and overcorrection for observed or unobserved confounders. When confirming biological pathways, the significance threshold imposed on the *p*-value may be less stringent than in studies formulating new hypotheses about epigenetic modifications following in utero exposure to maternal tobacco smoke [166]. In addition, statistical power depends not only on sample size but also on the precision of the studied exposures. Measurement errors can indeed cause large decreases in statistical power to detect associations. Therefore, specific attention should be paid to the collection and coding of the smoking status variable in order to minimize measurement errors. To further minimize false-positive and isolated CpGs, local approaches identifying differentially methylated regions have been proposed [167]. Several algorithms to define differentially methylated regions have been developed (reviewed by Robinson et al. [168]), among which Comb-p [169] and the recent ipDMR are suggested to be among the most efficient ones [170,171].

### 4.5. Meta-Analyses and Consortium Studies

Meta-analyzing results from different studies, despite difficulties for comparing measures of tobacco smoking during pregnancy and DNA methylation levels across studies, can lead to a significant gain in statistical power as well as a potential reduction of false-positive associations and selection bias compared to individual studies. In addition to the results from one study conducted on cord blood DNAm from 13 cohorts (*n* = 6685) gathered in the Pregnancy and Childhood Epigenetics (PACE) consortium [76] and a more recent one on placenta DNAm [66], few meta-analyses were conducted. Meta-analyzing results from 2821 individuals belonging to five prospective birth cohort studies, Wiklund and colleagues identified 69 differentially methylated CpGs in 36 genomic regions of blood DNA associated with maternal tobacco smoking during pregnancy, including one having a mediating effect on later child risk of schizophrenia [126]. Furthermore, using meta-analyses could be interesting when studying underrepresented subpopulations such as pre-term or very pre-term births. Such consortium efforts allow overcoming some of the limitations mentioned above, and they are a complementary approach to individual studies. In fact, consortia often include heterogeneous studies and do not permit investigating refined scientific questions. However, it is interesting to note that some consortia, such as PACE, use the pipeline of analysis common to all studies included in the meta-analysis, which guarantees a certain level of homogeneity in cell heterogeneity estimates, selection of confounders, and statistical methods to assess the association of DNAm with the exposure or outcome of interest.

### 4.6. Mediation Analyses

DNA methylation modification that is CpG site-specific has been found to mediate the relationships between maternal tobacco during pregnancy and offspring low birthweight [88] as well as later risk of schizophrenia [126]. Other studies did not show any mediation of maternal tobacco smoking during pregnancy by candidate genes, such as *NR3C1*, on child’s internal and external symptoms [172]. Despite a growing number of DNA methylation association studies, evidence of epigenetic mediation of exposure–outcome relationships remains sparse. This is partly due to statistical difficulties with analyzing data in large dimension [173]. The mediation techniques used for a single mediator cannot be generalized in a straightforward manner to high-dimension mediation. Mediation analyses usually report the mediated effects for each mediator separately, which is not informative regarding the overall mediated effect. In fact, methods considering each potential mediator separately do not allow efficient identification of the indirect effects when mutual influences exist among the mediators [174]. Therefore, mediation results considering each CpG independently and estimating the indirect effect separately from the other CpGs (or mediators) should be interpreted with caution. Given the complex causal structure of high-dimension data, analysis of high-dimension mediation requires caution and efforts to incorporate biological knowledge that validate causal inference.

## 5. Conclusions

Given the evidence of long-term effects of maternal tobacco smoking during pregnancy on offspring health, early epigenetic changes are likely to play a role in the etiology of these health issues. In particular, DNA methylation has been proven to be sensitive to environmental exposures and to be relatively stable over time, even in the absence of the initiating cause.

There is remarkable evidence suggesting an impact of maternal tobacco smoking during pregnancy on DNA methylation in different tissues, the most documented being cord blood, placenta, and children blood. Increasing evidence indicate lasting effects and postnatal persistence of maternal smoking associated alterations. A certain level of reversibility in DNAm upon smoking cessation before or during pregnancy is also suggested. Investigations of miRNA and downstream gene regulation are growing, while studies investigating histone modifications are still sparse. The incorporation of these approaches is needed to provide a more comprehensive picture of maternal smoking during pregnancy impact on epigenetic machinery and later health consequences, including potential transgenerational effects.

Epigenetic changes can serve as biomarkers of past exposure and biomarkers of early signs of diseases, which could be used for preventing the development of diseases. Research has made tremendous progress in the use of epigenetic markers provided by high-throughput technologies, in particular in association with prenatal smoking. However, there are still huge gaps to fill in our understanding on the role of maternal smoking during pregnancy on epigenetics. In particular, research needs to better address the dose–response relationship between maternal tobacco smoking and DNAm as well as the role of timing and duration of exposure. Cell and tissue specificities should be further explored. As such, the placenta seems to play a key role in prenatal programming and should be considered as deemed relevant for epigenetic epidemiological studies. Correlations of epigenetic variations between tissues will provide crucial information regarding the relevance of some tissues to be used as markers of maternal smoking during pregnancy.

Epigenetic markers are relevant candidates for mechanisms underpinning the programming of chronic diseases and developmental issues associated with maternal smoking during pregnancy. Yet few studies suggest the mediation of maternal tobacco smoking health effects through DNA methylation. The complex causal structure of high-dimension data implies huge statistical challenges to address the question of high-dimension mediation. Technical progress to make histone marks and miRNA more accessible to epidemiological research, combined with advancements in bioinformatic and biostatistic approaches for analyzing complex data, will provide new discoveries to elucidate our understanding of effects of maternal smoking during pregnancy.
